# Highly Sensitive H_2_S Sensor Based on the Metal-Catalyzed SnO_2_ Nanocolumns Fabricated by Glancing Angle Deposition

**DOI:** 10.3390/s150715468

**Published:** 2015-06-30

**Authors:** Kwang Soo Yoo, Soo Deok Han, Hi Gyu Moon, Seok-Jin Yoon, Chong-Yun Kang

**Affiliations:** 1 Department of Materials Science and Engineering, University of Seoul, 163, Seoulsiripdae-ro, Dongdaemun-gu, Seoul 130-743, Korea; 2 Center for Electronic Materials, Korea Institute of Science and Technology, 5, Hwarang-ro 14-gil, Seongbuk-gu, Seoul 136-791, Korea ; E-Mails: 113341@kist.re.kr (S.D.H.); moonhigyu@gmail.com (H.G.M); sjyoon@kist.re.kr (S.-J.Y.); 3 KU-KIST Graduate School of Converging Science and Technology, Korea University, 145, Anam-ro, Seongbuk-gu, Seoul 136-701, Korea

**Keywords:** H_2_S sensor, nanosensor, SnO_2_ nanocolumn, glancing angle deposition

## Abstract

As highly sensitive H_2_S gas sensors, Au- and Ag-catalyzed SnO_2_ thin films with morphology-controlled nanostructures were fabricated by using e-beam evaporation in combination with the glancing angle deposition (GAD) technique. After annealing at 500 °C for 40 h, the sensors showed a polycrystalline phase with a porous, tilted columnar nanostructure. The gas sensitivities (*S* = *R_gas_/R_air_*) of Au and Ag-catalyzed SnO_2_ sensors fabricated by the GAD process were 0.009 and 0.015, respectively, under 5 ppm H_2_S at 300 °C, and the 90% response time was approximately 5 s. These sensors showed excellent sensitivities compared with the SnO_2_ thin film sensors that were deposited normally (glancing angle = 0°, *S* = 0.48).

## 1. Introduction

Gas sensors have been used as detection devices for various gases in air. In particular, semiconductor gas sensors have proven to be very promising for monitoring the emission of gaseous species, and they represent a low-cost option compared to the standardized methods for ambient air classification, which require expensive and bulky equipment [1]. The gas-sensing mechanism of the semiconductor gas sensors is based on the resistance change of the sensor from the adsorption and desorption of the gases when specific gases interact with its surface [2]. Among the semiconducting metal oxides that are used for gas sensor applications, SnO_2_, *n*-type semiconducting metal oxide is the most widely studied material because it is sensitive to various gaseous species. In addition, gas sensors based on ZnO, In_2_O_3_, WO_3_, TiO_2_, Fe_2_O_3_, and others have also been investigated [3,4,5,6,7]. Especially, catalyzed SnO_2_ nanorod or nanowire-like structures were used as gas sensors with high sensitivities [8,9].

In terms of their application as hydrogen sulfide (H_2_S) gas sensors, the sensors based on SnO_2_ [10,11], WO_3_ [1,12], Fe_2_O_3_ [13], BaTiO_3_ [14], CuO-SnO_2_ composites [15,16,17,18], and CuO-WO_3_ composites [19] have been extensively studied. In these cases, many techniques have been used to study the adsorption and decomposition of H_2_S on the surface of metals such as Ag, Au, Pd, and Rh [20]; when Au films were used as the H_2_S sensor, their sensitivity was very low [21]. In addition, options including a chemiluminescent sensor and a colorimetric sensor, have been suggested as H_2_S sensors [22,23], and sensor-based methods for monitoring H_2_S and recent developments in H_2_S-sensing instrumentation were systematically reviewed [24].

H_2_S is widely used in various chemical industries and research laboratories, and is a very poisonous, corrosive, flammable, and explosive gas with the characteristic foul odor of rotten eggs. Exposure to lower concentrations can result in eye irritation, a sore throat and cough, nausea, shortness of breath, and fluid in the lungs. 10 ppm is the Occupational Safety and Health Administration (OSHA) permissible exposure limit (8 h time-weighted average) and 20 ppm is the acceptable ceiling concentration established by OOSHA [25]. H_2_S such as NO and CO is also produced in small amounts by some cells of the mammalian body and has a number of biological signaling functions. Accordingly, an accurate measurement and control of the H_2_S gases of low concentrations is very important to protect human lives.

In the present study, to enhance H_2_S gas-sensing characteristics, Au- and Ag-catalyzed SnO_2_ thin films with morphology-controlled nanostructures were fabricated by using e-beam evaporation in combination with the glancing angle deposition (GAD) technique. The GAD technique produces films with a high surface area that consist of isolated columns and provide excellent control over film/column morphology [26]. Use of e-beam GAD provides a method to control nanostructure unlike chemical vapor deposition, which is a non-line of sight deposition technique. By manipulating the tilt angles of the substrates and reaction-chamber oxygen pressures, it is possible to achieve a structural evolution from two-dimensional columnar films to one-dimensional nanorods, composites of nanorods and nanoparticles, and zero-dimensional nanoparticles [27,28]. X-ray diffraction (XRD) was used to analyze the films’ crystalline structure and the microstructures were observed using field emission scanning electron microscopy (FESEM). The sensors’ H_2_S sensing properties such as sensitivity and response time were subsequently measured and evaluated.

## 2. Experimental Procedure 

To fabricate nanostructured sensors with a high reproducibility and easy mass production, metal-catalyzed SnO_2_ nanocolumns were deposited using the GAD technique.

**Figure 1:**
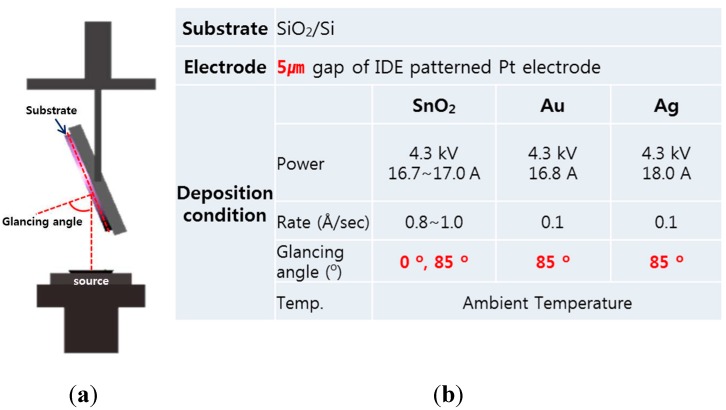
(**a**) A schematic diagram of the e-beam evaporation using the glancing angle deposition technique; (**b**) Deposition condition of the sensors.

The substrate used for these sensors was the SiO_2_/Si wafer with Pt/Ti interdigitated electrodes (IDEs) that is comprised of an inter-electrode gap of 5 µm. The thicknesses of the Pt/Ti IDEs were 100 nm/50 nm, respectively, and IDEs were fabricated by photolithography and dry etching. SnO_2_ thin films of a 100 nm thickness (subsequently referred to as “SnO_2_ thin film sensor”) were deposited onto the substrate at an ambient temperature using an e-beam evaporator under 2 × 10^−5^ Torr. In the same manner, SnO_2_ thin films with nanocolumn morphology using the GAD technique (subsequently referred to as “SnO_2_ nanocolumn sensor”) were evaporated at a glancing angle of 85°, based on previous research [29]. The glancing vapor flux of atom randomly forms an initial nucleus like a nanoisland on the substrate. The initial nucleus makes a self-shadowed region that is not deposited on the opposite side of vapor flux. Next, continually growing up along one side by glancing vapor, the porous nanocolumns are formed on the tilt. In addition, Au or Ag of a 5 nm thickness was deposited onto the SnO_2_ nanocolumns. A schematic diagram of the e-beam evaporation as a function of the incident angle using the GAD technique and the details of the deposition conditions are shown in Figure 1. After the deposition, these thin films and nanocolumns were annealed at 500 °C for 40 h in air to produce SnO_2_ polycrystalline and metal nanoislands by aggregation.

The crystallinity and phase of the SnO_2_ films were characterized by a glancing angle X-ray diffractometer (D/Max-2500, Rikagu) with a 2θ scan from 20° to 80°, where Cu K_α_ (λ = 1.5414 Å) radiation was used for the X-ray source and the incident angle was fixed at 2°. The morphologies of the SnO_2_ films’ surfaces and cross-section were observed using an FESEM (FEI Inspect F50) operating at 8 kV.

The responses of these sensors to H_2_S gas were tested at 300 °C and changes of the sensor resistance were monitored during the transition from dry air to 5 ppm H_2_S gas balanced with N_2_ gas. To eliminate any interfering effects, a constant flow rate of 500 sccm was used for both the dry air and H_2_S gas conditions. Sensor resistances were measured at a DC bias voltage of 0.5 V using a source meter (Keithley 2400). Lastly, the H_2_S sensitivity and response time of the sensors were discussed.

## 3. Results and Discussion

### 3.1. Crystal Structure and Microstructures

**Figure 2:**
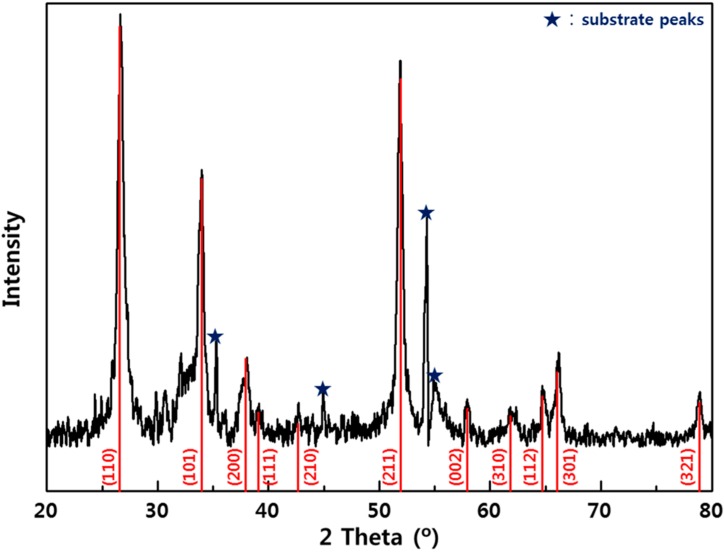
X-ray diffraction pattern of the SnO_2_ thin film.

The XRD pattern of the SnO_2_ films is shown in Figure 2, and confirming that the as-deposited films were amorphous and the films annealed at 500 °C for 4 h were polycrystalline. The XRD peaks with a SnO_2_ tetragonal phase corresponded well with the JCPDS 41-1445 [30].

**Figure 3:**
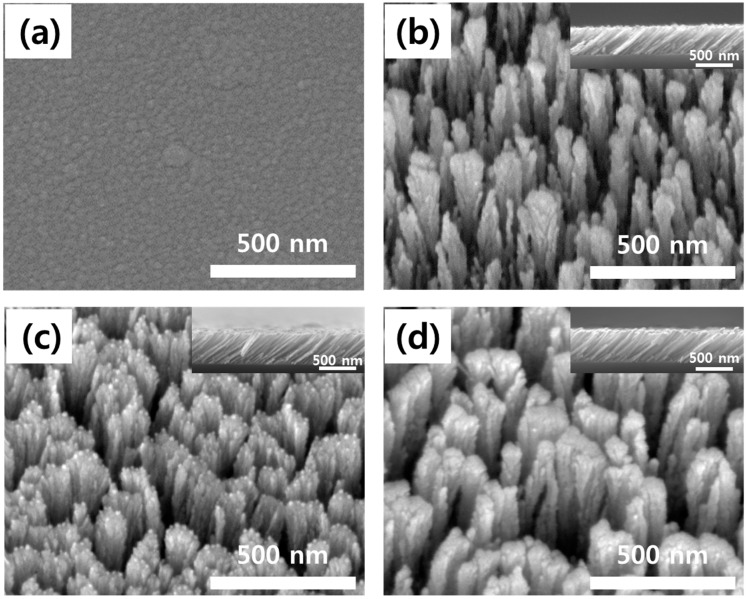
FESEM images. (**a**) Surface of SnO_2_ thin film (glancing angle = 0°); (**b**) Surface and cross-section of SnO_2_ thin film (glancing angle = 85°); (**c**) Surface and cross-section of Au-catalyzed SnO_2_ thin film (glancing angle = 85°); (**d**) Surface and cross-section of Ag-catalyzed SnO_2_ thin film (glancing angle = 85°).

Surface and cross-sectional FESEM images of the SnO_2_ films are shown in Figure 3. Figure 3a shows an image of the SnO_2_ thin film (the film deposited at a glancing angle of 0°). The SnO_2_ thin film was relatively dense and was composed of a range of nanometer-sized grains. Alternatively, Figure 3b shows images of the SnO_2_ film deposited at a glancing angle of 85°, whereby its surface microstructure was nanoporous and a cross-sectional view revealed a tilted columnar structure. These results indicate that the nanocolumn sensors have larger sensor/gas contact areas than the thin film sensor of Figure 3a. As shown in Figure 3c,d, in the case of the Au- and Ag-catalyzed SnO_2_ nanocolumn sensors, the Au and Ag aggregated on the nanocolumn surface during the annealing process and a catalytic nanoisland was therefore formed.

### 3.2. Gas-Sensing Properties

Gas sensitivity (*S*) is defined here as *R_gas_/R_air_*, where *R_gas_* is the resistance of the sensor in the H_2_S gas and *R_air_* is its resistance in dry air.

At temperatures of 200–500 °C, metal oxides with *n*-type carrier paths, such as SnO_2_, ZnO, In_2_O_3_, and WO_3_, primarily respond to oxidizable gases such as H_2_, CH_4_, CO, and H_2_S, thereby increasing their conductivity [2]. It is well known that the surfaces of oxide semiconductors can adsorb oxygen from the ambient atmosphere. The chemisorbed oxygen species act as surface acceptors, trapping electrons and increasing the resistance of the metal oxides, as follows [31]:

(1) $$O_{2}\left(ads\right)+\;e^{-}\;↔\;O_{2}^{-}\left(ads\right)$$

(2) $$O_{2}^{-}\left(ads\right)+\;e^{-}\;↔\;2O^{-}\left(ads\right)$$

(3) $$O^{-}\left(ads\right)+\;e^{-}\;↔\;O^{2-}\left(ads\right)$$

The gas-sensing mechanism of the H_2_S gas can be explained by the reaction of the H_2_S on the SnO_2_ film in accordance with Equation (4); that is, the H_2_S rapidly reacts with the adsorbed oxygen and therefore releases the captured electrons back into the bulk. The surface reactions between the H_2_S and the oxygen species can be described in the following manner [31]:

(4) $$H_{2}S+3O^{2-}\left(ads\right)\;\to \;H_{2}O\left(g\right)+SO_{2}\left(g\right)+6e^{-}$$

The sensor resistance will therefore decrease when the SnO_2_ sensors are exposed to the H_2_S gas.

**Figure 4:**
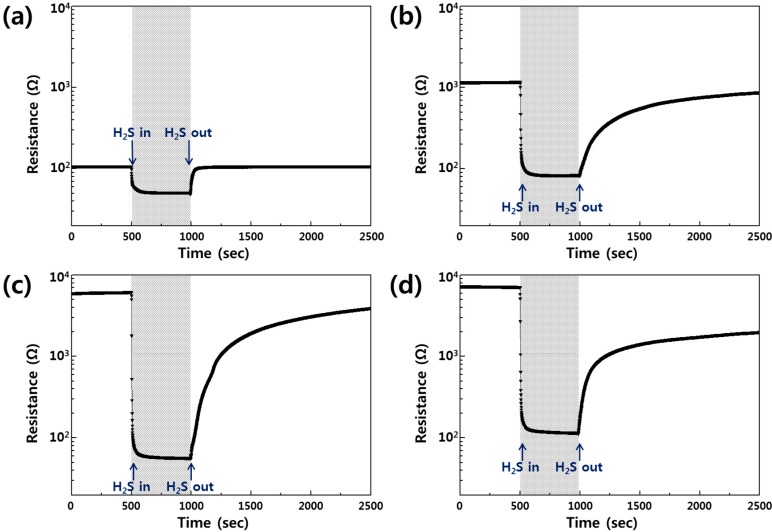
Gas-sensing properties of the sensors exposed to 5 ppm H_2_S at 300 °C. (**a**) SnO_2_ thin film sensor; (**b**) SnO_2_ nanocolumn sensor; (**c**) Au-catalyzed SnO_2_ nanocolumn sensor; (**d**) Ag-catalyzed SnO_2_ nanocolumn sensor.

Figure 4 shows the H_2_S gas-sensing properties of the SnO_2_ sensors at an operating temperature of 300 °C and their sensitivities are summarized in Table 1. As shown in Figure 4a, the resistance of the SnO_2_ thin film sensor in dry air was 107.57 Ω (*R_air_*), but its resistance when it was exposed to the 5 ppm H_2_S gas decreased to 51.23 Ω (*R_gas_*), resulting in a gas sensitivity (*S* = *R_gas_/R_air_*) of 0.48. This change can be explained by Equation (4), and the result is similar to those from previous studies [32].

**Table 1:** Resistance and H_2_S sensitivity of the sensors measured at 300 °C.

	SnO_2_ Thin Film	SnO_2_ Nanocolumn	Au-Catalyzed SnO_2_ Anocolumn	Ag-Catalyzed SnO_2_ Anocolumn
*R_air_* (Ω)	107.57	1176.1	6209.5	7170.2
R_gas_^*^ (Ω)	51.23	83.9	56.87	111.9
*S* = *R_gas_* /*R_air_*	0.48	0.071	0.009	0.015

R_gas_^*^ = sensor resistance in 5 ppm H_2_S.

However, in the case of the SnO_2_ nanocolumn sensor fabricated by the GAD technique, the base resistance of the sensor in dry air had relatively high values that are approximately 10 times greater (*R_air_* = 1176.1 Ω), and its H_2_S sensitivity was significantly enhanced (*S* = 0.071), as shown in Figure 4b; the SnO_2_ nanocolumn sensor shows very narrow and long, tilted columns with diameters of a few nanometers. This nanostructured shape increased the specific surface area that could react with gas, and adsorption of ionized oxygen species (O^−^, O_2_^−^, O^2−^) changed many regions of narrow necks between nanocolumns into the full depletion area (space charge layer); therefore, the resistance of the SnO_2_ nanocolumn sensor abruptly increased [29]. In the previous work, it was reported that the surface area of WO_3_ nanocolumnar thin film was about 32 times larger than that of a plain WO_3_ thin film by Brunauer-Emmett-Teller (BET) measurements [29]. This result shows which the increased surface area that can react to the gas will enhance the gas sensitivity. Moreover, it is also noted that the nano-sized narrow necks (10~30 nm) between the nanocolumns have a strong effect on the gas response [29]. Because the double Schottky barrier heights in the intergrain boundaries which are induced in the narrow neck region significantly increase, the sensitivity is dramatically enhanced by the change of conductance during the gas reaction [29]. We believe that these unique nanostructures as well as the increased surface area have an effect on the gas sensitivity.

The Au- and Ag-catalyzed SnO_2_ nanocolumn sensors had higher base resistances (*R_air_* = 6209.5 Ω and 7171.6 Ω, respectively), as shown in Figure 4c,d. These higher values are due to the large depletion area that was formed by the trapping of electrons around the Au and Ag nanoislands, whose sensitivities were 0.009 and 0.015, respectively. As shown in Table 1, the base resistance of the Ag-SnO_2_ sensor is higher than that of the Au-SnO_2_ sensor, while the resistance change of the Ag-SnO_2_ sensor is lower and, therefore, its sensitivity has a higher value. It is assumed that the size of the Ag catalyst is relatively small compared to that of the Au catalyst and the Ag nanoislands cover the SnO_2_ nanocolumn surface; therefore, the reaction area of the nanocolumnar SnO_2_ with the H_2_S gas was decreased by the regions overspread with Ag nanoislands, which is called “catalytic filtering effect” [33].

Compared with the SnO_2_ thin film sensor that was fabricated normally with an e-beam evaporator, the sensitivity of the Au-catalyzed SnO_2_ nanocolumn sensor to 5 ppm H_2_S gas was enhanced 53 times.

The dynamic response and recovery curves of the sensors are also shown in Figure 4. When the H_2_S gas was injected, the sensors exhibited a fast response and a moderately slow recovery. The 90% response times of the sensors were approximately 41 s, 7 s, 5 s, and 6 s for Figure 4 a–d.

## 4. Conclusions

In the present study, e-beam evaporation was used in combination with the GAD technique to prepare highly sensitive H_2_S sensors. The gas-sensing material, SnO_2_ formed a polycrystalline tetragonal phase after annealing at 500 °C for 40 h. The surface morphology of the sensors that were fabricated by the GAD technique showed a porous, tilted columnar nanostructure. The Au-catalyzed SnO_2_ nanocolumn sensor showed an excellent sensitivity (*S* = 0.009 to the 5 ppm H_2_S at 300 °C) and a rapid response. We consider that the metal-catalyzed SnO_2_ nanocolumn sensors fabricated using the GAD process could be effectively used to detect low levels of H_2_S gas.
